# Process evaluation of an integrated community-based intervention for promoting health equity in children in a new residential development area

**DOI:** 10.1186/s13690-024-01246-z

**Published:** 2024-02-06

**Authors:** Stephan Voss, Julia Bauer, Caroline Jung-Sievers, Graham Moore, Eva Rehfuess, Valerie Zu Rhein, Michaela Coenen

**Affiliations:** 1 Chair of Public Health and Health Services Research, Institute for Medical Information Processing, Biometry, and Epidemiology (IBE), Faculty of Medicine, LMU Munich, Elisabeth-Winterhalter-Weg 6, Munich, 81377 Germany; 2 Pettenkofer School of Public Health, Munich, Germany; 3 Centre for Development, Evaluation, Complexity and Implementation in Public Health Improvement (DECIPHer), School of Social Sciences, Cardiff University, Cardiff, UK; 4 Wolfson Centre for Young People’s Mental Health, Cardiff University, Cardiff, UK

**Keywords:** Child and adolescent health, Integrated community-based intervention, Health promotion, Health equity, Public health service, Process evaluation, Mixed methods

## Abstract

**Background:**

Reducing health inequities for children from a disadvantaged background is an important task in public health. While intersectoral partnerships are a promising way to achieve this, few studies have examined the factors influencing the success of these interventions. In this study, we conducted a process evaluation of the integrated community-based intervention Präventionskette Freiham that the city of Munich, Germany, has implemented in a new residential development area. The aim was to investigate the implementation process as well as barriers and facilitators.

**Methods:**

Following a mixed methods approach, we collected data from different core groups making up Präventionskette Freiham from April 2020 to August 2022, exploring their perspective on the implementation process. We conducted repeated qualitative interviews with the network coordinators and eleven local professionals from institutions engaged with or relevant for the intervention. We also undertook a focus group with four members of the advisory group representing the three municipal departments guiding the intervention. Ego-centered network maps were drawn by the network coordinators to chart the development of the network. Subsequently, we also conducted an online survey with local network members.

**Results:**

At the early stage of the implementation process, the intervention was able to integrate actors from different sectors, serving as a platform for mutual exchange. However, the network produced limited output. According to the interviews, this may be mainly attributable to the early development status of the area. We identified seven topics that may act as facilitators or barriers to implementation of Präventionskette Freiham: (1) availability of resources, (2) political and administrative support, (3) the network coordinators, (4) network-internal processes, (5) trans-institutional cooperation, (6) perceived benefits of engagement, and (7) the output of the network.

**Conclusions:**

The early development status of the area was a challenge for the intervention. This emphasizes the need to carefully consider context when planning and implementing integrated community-based public health interventions in new residential development areas.

**Supplementary Information:**

The online version contains supplementary material available at 10.1186/s13690-024-01246-z.

**Table:** 

Text box 1. Contributions to the literature
• We conducted a process evaluation of a public health intervention that aims to decrease health inequities in children in a new residential development area in Munich, Germany.
• We identified several factors influencing implementation, mainly resources, political and administrative support and benefits of engagement perceived by network members. The absence of institutions and residents in the new area hindered the implementation of the network.
• This is the first study to examine facilitators and barriers to the implementation of a public health intervention in a new residential development area. Results may guide the planning and implementation of similar interventions.

## Background

Children and adolescents from socioeconomically disadvantaged families and deprived neighbourhoods tend to grow up with worse health and impaired development compared to their peers. This association has been observed in Germany [1] as well as in other countries worldwide [2–4]. These inequities can manifest in various outcomes related to health and development such as low birth weight, anxiety disorders, obesity, hypertension and delayed motoric or cognitive development [2, 5]. Furthermore, children from low-income households are more often absent from school, widening the gap to their peers regarding health and education [6, 7]. Disadvantages emerging during pregnancy or childhood tend to persist at the transition from school to work or to university [8] and into later stages of adulthood [9]. Therefore, providing children and adolescents with equal opportunities to grow up healthy and without socioeconomic disadvantage is not only required from an ethical point of view, but can also be considered as an approach to reduce inequities across the life-course and to improve the health status in the population overall [10].

In Germany, municipal public health strategies that aim to reduce inequities in children and adolescents are known as Präventionsketten (literal translation: “prevention chains”) [11]. The goal of these integrated community-based interventions is to create a network of stakeholders from different sectors relevant for child health and development, mainly health, education and social services. The approach aims to promote a more coordinated collaboration between these stakeholders that facilitates an improved health, educational and social services infrastructure. The resulting infrastructure is intended to be more accessible and adequate for the needs of children at different stages of their development, especially from a low socio-economic background [12]. Characteristic of Präventionsketten is the emphasis on transitions, e.g. from kindergarten to elementary school [12]. Transitions can be both opportunities and stressors for child development [13], and may pose particular challenges for children from a lower socioeconomic background [14]. Therefore, supporting children going through these transitions may be a means to tackle health inequities.

In 2016, the city of Munich in Southern Germany started to build a new residential development area named Freiham for approximately 25,000 citizens at its southwestern border. Simultaneously, the municipal administration decided to implement the intervention Präventionskette Freiham in the new district. The goal was to build an intersectoral network to ensure a better support infrastructure for children and their families in order to promote health, access to education, social participation and health equity from the very beginning [15]. Implementation in the Freiham district started in early 2020, shortly after the first residents arrived in late 2019.

A recent systematic review indicates that community-based interventions have the potential to reduce health inequities, mainly through synergistic effects between the different components of the interventions [16]. Intersectoral approaches seem to be able to improve the health and development status off children and adolescents with socioeconomic disadvantages mainly by building networks, improving services and increasing access to support and information [17]. As interventions such as Präventionskette Freiham can be considered as complex interventions [18], their effectiveness should be evaluated carefully, taking into consideration the specific implementation context. Complex interventions usually feature multiple components, include multiple groups at differing organizational levels and operate through multiple, often long causal pathways [19]. Additionally, as “events in systems” [20], interventions are not only affected by the context they are implemented in, but the context may adapt and change due to the intervention [21, 22]. Measuring only outcomes is usually not enough for evaluating these interventions, because this approach can only assess whether an intervention is successful, but not how and why; if unsuccessful, it remains unclear whether this is due to the intervention itself, the way it is being implemented or contextual aspects [23]. Moreover, considering that complex interventions are part of a complex system, it is challenging to assess whether a change occurred because of the intervention or due to other factors within the system. Conducting a process evaluation can help to obtain a deeper understanding of how and why an intervention works in a specific context – or, likewise, why it fails to reach its goals [24]. The insights gained can be used to refine the intervention itself and to explore whether and how the intervention may be transferred to other contexts [22].

To the best of our knowledge, a process evaluation of an integrated community-based intervention in the specific context of a new residential development area has not been conducted to date. Typically, interventions involve introducing something new into an established complex system. Therefore, investigating the implementation of Präventionskette Freiham provides an important opportunity to research how an intervention co-evolves within an evolving context that it is supposed to build from the outset, and how intervention and context interact with each other. This paper describes a process evaluation of the intervention Präventionskette Freiham using a mixed methods design. This aimed to answer the following questions: (i) How did the implementation process evolve during the research period? (ii) What were facilitators and barriers to implementation?

## Methods

### Setting

Freiham is a new residential development area with a size of 190 hectares (1.9 square kilometers) that the city of Munich, Germany, is currently building at its southwestern border. Construction started in 2016. The first residents arrived in late 2019. After completion, approximately in the year 2040, 25,000 citizens are expected to live in the area [25]. Due to a high proportion of social housing, many families with young children and a low socioeconomic background are expected to live in Freiham [15], suggesting an increased need for a supporting infrastructure regarding health, education and social services for these target groups. According to data provided to the research team by the Munich registry office, 368 households with children aged 18 or below were living in Freiham in December 2021.

### Intervention

Präventionskette Freiham was initiated in 2015 by three municipal departments of Munich: The Department of Health, the Department of Social Services and the Department of Education and Sports. It aims to create an intersectoral network of local professionals and institutions in the district of Freiham to promote health, increase access to education and social participation and diminish health inequalities in children and adolescents. The Präventionskette comprises four core groups: (a) A steering committee, which consists of the heads of the three involved municipal departments and meets twice per year to decide on strategic developments. (b) An advisory group, which consists of members of the three departments involved in the intervention as well as the network coordinators, and meets monthly to discuss next steps for the implementation. (c) The network coordinators, who are responsible for building and coordinating the local network in the district of Freiham and for communicating between the municipal administration and local actors. Until August 2020, this position consisted of one person. After that, the position was shared by two persons. (d) The local network in Freiham, which consists of the network coordinators and professionals from the health, education, and social services sectors responsible for working with children and adolescents in the district of Freiham. As a “production network”, the task is to refine the existing support infrastructure for children and families and to develop specific projects that respond to the existing needs of the target groups. These would be referred to as outputs of the network. Members of the local network meet during regularly scheduled working group meetings to exchange information and to decide on priority activities in a participatory approach. There are two distinct working groups for the local professionals, according to the ages of the children they target (0-6 years, 6-17 years). Building the local network started in early 2020, parallel to the start of the COVID-19 pandemic.

### Study design

This process evaluation is part of the evaluation of the intervention Präventionskette Freiham that is conducted by the Chair of Public Health and Health Services Research at LMU Munich. We applied a concurrent triangulation mixed methods design [26], with a strong emphasis on qualitative methods. Data of the different approaches were collected and analyzed separately, and integrated narratively when interpreting findings [27]. Most of the process evaluation of Präventionskette Freiham took place from November 2019 to December 2021.

**Fig. 1:**
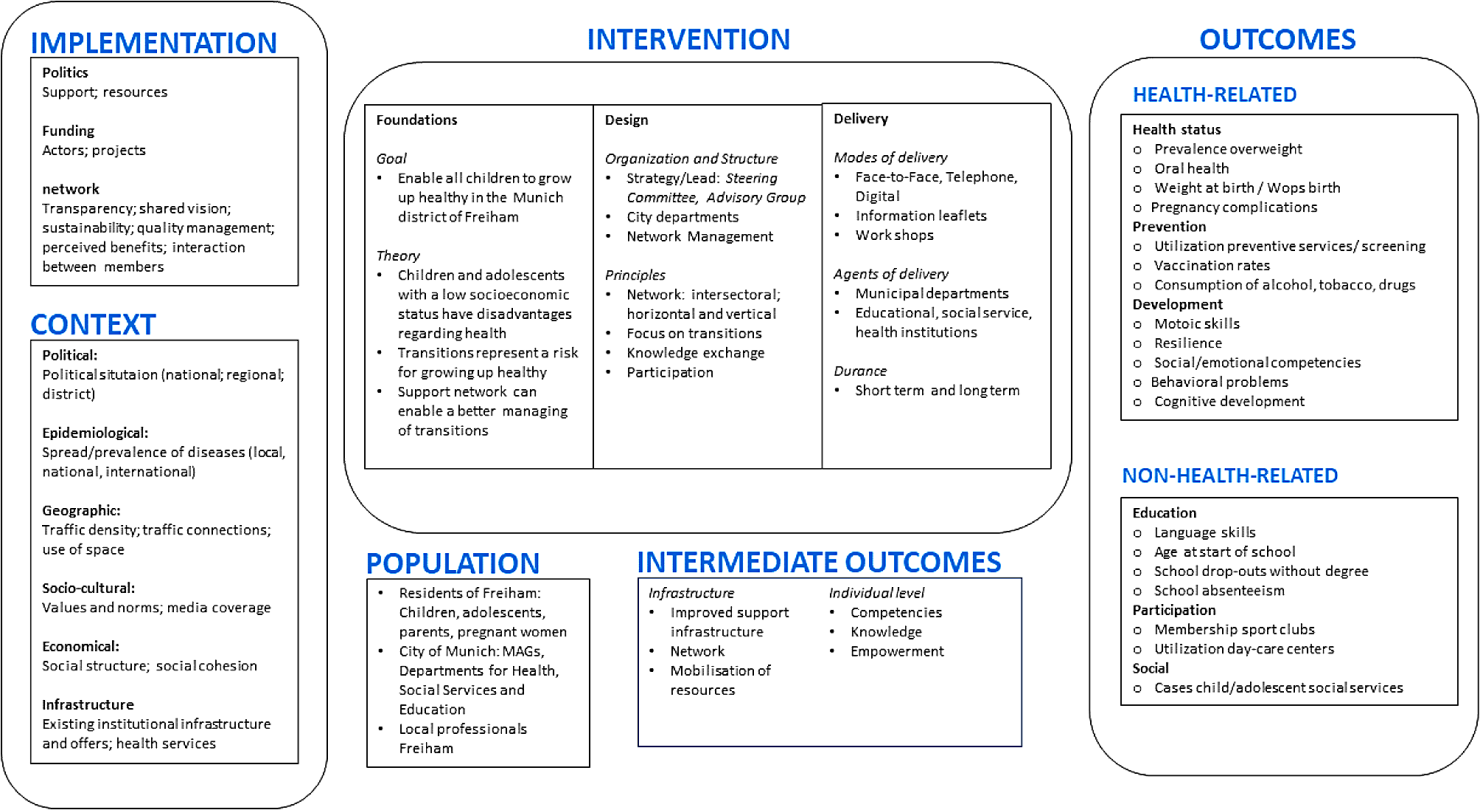
Initial logic model of the intervention Präventionskette Freiham developed at the beginning of the process evaluation

Prior to the process evaluation, a network analysis with members of the advisory group of Präventionskette Freiham explored their expectations regarding the implementation of the network [28] and a qualitative interview study with representatives of Präventionsketten in multiple German municipalities investigated the perspectives of the participants on facilitators and barriers to the work of Präventionsketten [29]. Additionally, an initial logic model was developed that aimed to present all core elements of the intervention (Fig. 1). The model aimed to support developing insights into the mechanisms underlying the intervention and generating hypotheses for the research process. We chose a system-based logic model approach to highlight the role of the context the intervention is implemented in [30]. The logic model was based on official documents of Präventionskette Freiham, literature searches on similar integrated community-based interventions for children and adolescents, and considerations within the research team. Since January 2022, the Chair of Public Health and Health Services Research has also been conducting an outcome evaluation of Präventionskette Freiham. Here, the goal is to set up a monitor that allows the long-term assessment of relevant outcomes in the Freiham district.

### Data collection

**Fig. 2:**
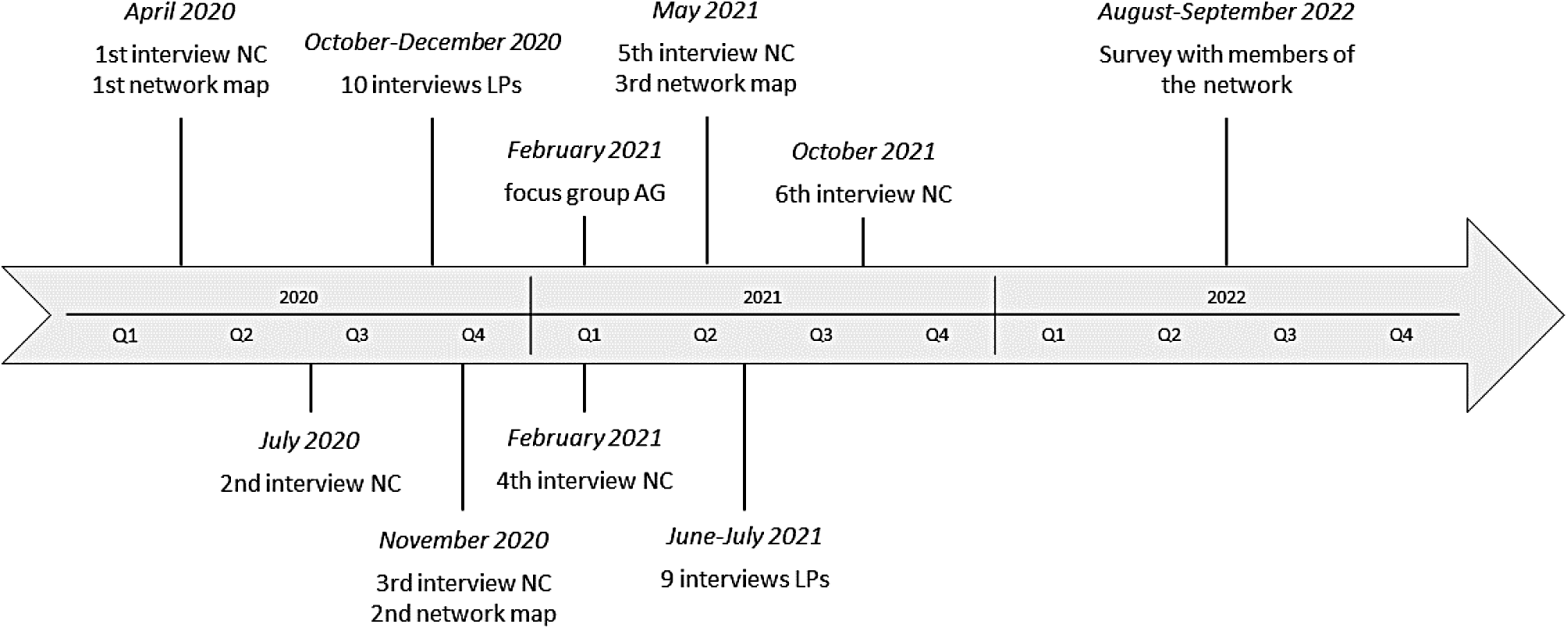
Data collection process for the process evaluation of präventionskette freiham (NC = network coordinator; LP = local professional; AG = advisory group)

The process evaluation consisted of four sub-studies (Fig. 2):

- Repeated expert interviews with the network coordinator every 3-4 months (April 2020-October 2021), to gain detailed insights on the development of the implementation process from the perspective of a key stakeholder. Additionally, an ego-centered network map was drawn at every second interview with the network coordinator to gain insights on the growth of the network and potential underrepresented sectors.
- One focus group with representatives of the three involved municipal departments of Präventionskette Freiham, who had all been involved with the project for many years through their activity in the advisory group. Again, the goal was to explore the implementation process from the perspectives of key stakeholders. The focus group took place in February 2021.
- Qualitative interviews with local professionals from relevant institutions for the intervention to explore their perspective on the implementation process and on facilitators and barriers for participating in the network. These could be located in Freiham or in surrounding districts, as long as Freiham was within their area of responsibility. Participants were interviewed twice, where possible (October-December 2020; June-July 2021).
- An online survey with members of the local network in Freiham to explore their perspective on working in the network and potential personal benefits (August-September 2022).

All sub-studies were conducted in compliance with the ethical standards of the Declaration of Helsinki [31] and each of them was approved individually by the ethics committee of the Faculty of Medicine at LMU Munich.

Potential participants for the qualitative interviews and the focus group were contacted by the first author (SV) via email to inquire about their general interest in participating. The contact details for the network coordinators and the representatives of the city departments had been known to the research team prior to the interviews and focus group. Email addresses of the local professionals were obtained via Google searches or via the network coordinators. For the focus group, we contacted all members of the advisory group of Präventionskette Freiham regarding participation. All these individuals were employees of one the three municipal departments of Munich (the Department of Health, the Department of Social Services and the Department of Education and Sports). For the interviews with local professionals, we used a purposeful sampling strategy. We aimed to represent all sectors relevant for the intervention (health, social services, education) and to cover the two distinct age groups of children targeted (0-6 years, 6-17 years). We recruited professionals from schools and other municipal educational institutions, professionals from social institutions working with families and adolescents or development supporting institutions as well as nurses working in the area. Professionals were eligible in case their institution was either located in Freiham or the surrounding area, as long as residents of Freiham were among their clients. Furthermore, while most of the interviewees had already participated in activities of the local network of Präventionskette Freiham, we also aimed to include some professionals who had not yet participated, to cover external perspectives on the network. There were no previous contacts between the research team and the professionals contacted for the interviews.

Where potential interviewees agreed to participate, they received detailed information about the study and the conditions for participating. Furthermore, directly before the interview or the focus group, they were informed once more by the interviewer (SV) about the detailed conditions. The interviewer only proceeded with data collection in case the participants gave their written informed consent.

Semi-structured interview guides were used for data collection. These were developed based on assumptions derived from the logic model. English translations of the interview guides can be found in Appendix 1. Interview guides contained questions exploring the interviewees’ opinions about their perceived role and the goals of Präventionskette Freiham, the network-related events they had participated in, their views on facilitators and barriers for the implementation process and challenges for the area. The interview guides were adapted for each sub-study. Due to the COVID-19 pandemic, the focus group and most of the interviews were conducted virtually, either via the open source software Jitsi Meet or via Webex. Three interviews were conducted face-to-face. Interviews were recorded with the open source software Audacity and transcribed verbatim with f4transcript [32]. Transcripts were sent to the interviewees by email for approval, and thereupon pseudonymized by replacing names of individuals with a 5-digit numeric code and names of institutions with a 2-digit numeric code.

At the end of every second interview with the network coordinator, an ego-centered network map based on Kahn and Antonucci [33] was drawn with the software VennMaker 2.0.2 [34]. As these interviews took place virtually, the interviewer shared his screen with the network map template. The interviewee was asked to name up to 20 of their most important collaborators within the network of Präventionskette Freiham, and to state where these actors belonged on the map within three concentric circles (inner circle: “strong cooperation”; medium circle: “medium cooperation”; outer circle: “weak cooperation”). The interviewer thus placed each indicated person on the network map. After the interview, the network map was sent to the interviewee via email to provide an opportunity for correcting mistakes or to add persons that might have been forgotten.

Interviews with the network coordinators were conducted approximatively every three months from April 2020 until October 2021, according to a pre-defined frequency. For the focus group with members of the advisory board, after analyzing the transcript of the focus group, we concluded that no relevant new information was likely to be derived from conducting another focus group. Likewise, we stopped recruiting new participants for the interviews with local professionals when we found that the main topics were being repeated in the conducted interviews and data saturation was likely to have been achieved.

The survey of members of the local network was conducted with a questionnaire using LimeSurvey [35]. All 67 network members were contacted via email by the network coordinators on 2nd August, 2022, and invited to participate in the survey. The deadline for participation was 30th September, 2022. Several weeks after the start of the survey, invited members received a reminder via email. An English translation of the survey is available in Appendix 2.

### Data analysis

**Fig. 3:**
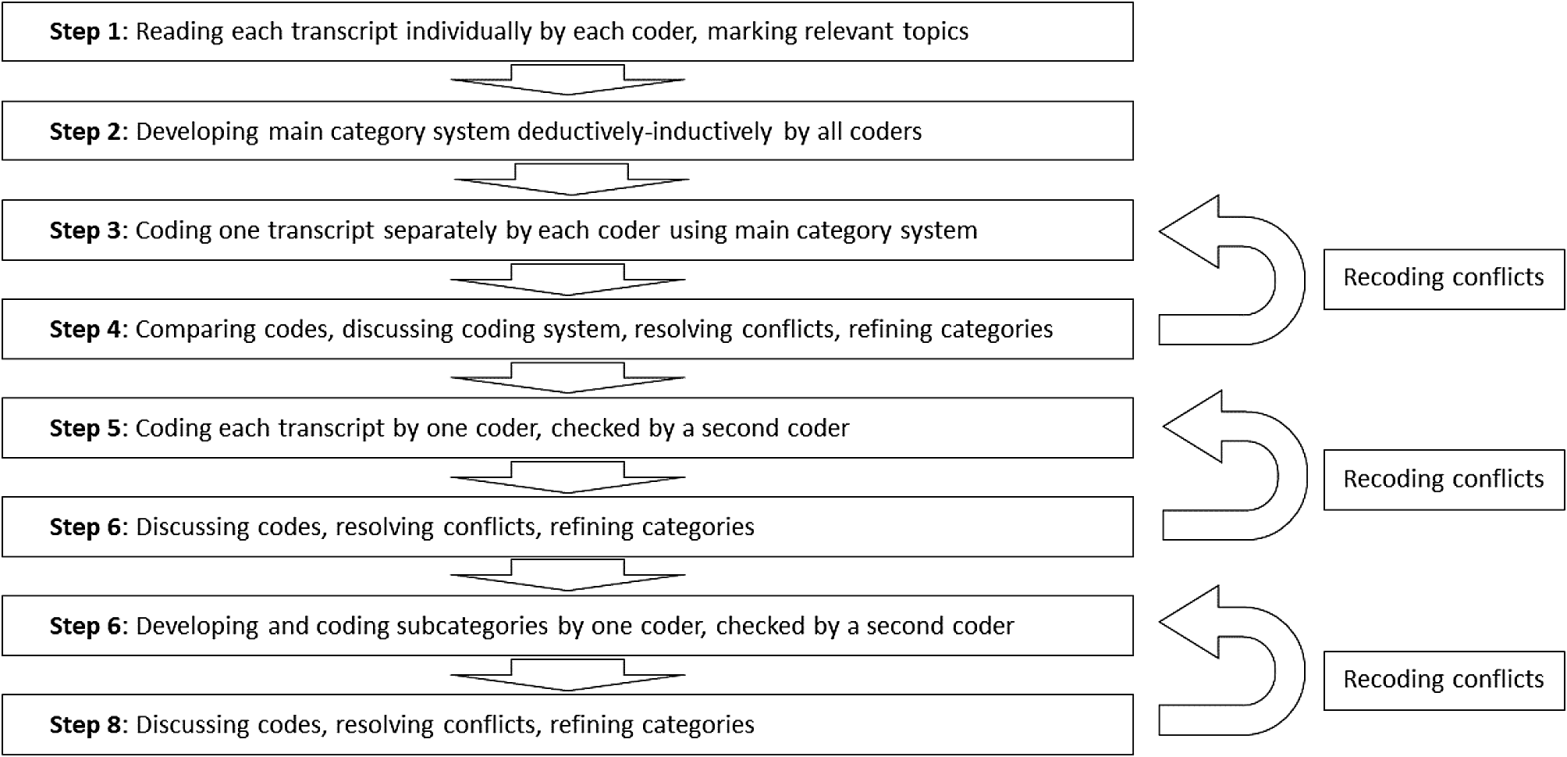
Step-by-step process of qualitative content analysis for the interviews and the focus group

The transcripts of the qualitative interviews and the focus group were analyzed using qualitative content analysis based on Mayring [36] by three coders (JB, SV, VZR) using MAXQDA [32] in a multistep iterative process (Fig. 3). The main category system was developed by each researcher coding the same interview independently and discussing changes to the category system based on disagreements, until the main categories were finalized. Furthermore, every coded interview was checked by a second team member. All disagreements during the coding process were discussed within the group and solved collectively. All quotations in this manuscript were translated verbatim by the first author and checked by a native speaker.

In the network maps, each actor was categorized into one of the three sectors: health sector, social services sector or educational sector. In cases where no clear attribution was possible, actors were categorized as “Other”. The network maps and the survey were analyzed descriptively with R 4.2.0 [37]. Answers to the open questions in the survey were analyzed using qualitative content analysis and the coding scheme developed for the interviews and focus group.

## Results

### Results for interviews and focus group

#### Participants

Six qualitative interviews were conducted with the network coordinators from April 2020 through to October 2021. After the first two interviews, in the summer of 2020, there was a change of personnel in the position of the network coordinator. Therefore, the last four interviews were conducted with one of the two new persons responsible for coordinating the network. The focus group with members of the advisory group took place in February 2021 with four participants. The Department of Social Services of the city of Munich was represented by two staff members, the Department of Health and the Department of Education and Sports by one each.

A total of 19 interviews were conducted with local professionals in Freiham or surrounding districts: ten in October-December 2020, nine in June-July 2021. Of these, seven professionals were interviewed at both times, three only October-December 2020, two only June-July 2021. One interviewee from October-December 2020 withdrew their informed consent after having read their transcript. Therefore, 18 interviews with eleven local professionals progressed to qualitative content analysis.

#### Implementation of the intervention

Most of the interviewees reported a general satisfaction with the way the network evolved. Especially in interviews in June-July 2021, local professionals stated that the network had become a reliable platform they could turn to with their matters, and that it was considered as such by other actors in the district, too. However, while most interviewed professionals generally agreed to the idea of Präventionskette Freiham being a network to support children and families, many were unsure about its specific tasks. The diversity of network members was considered an asset by the professionals, allowing them to gain new perspectives and to obtain information that would otherwise be unobtainable. However, it was criticized that there were only few professionals from the health and educational sector attending the working group meetings, despite the network coordinators trying to integrate these actors. In the interviews with professionals from these sectors, it became clear that the COVID-19 pandemic meant a lot of additional work for the professionals, limiting their capacities for networking. Especially professionals from schools were engaged with implementing pandemic control measures and professionals from the municipal health department had to work in COVID-19 task forces instead of pursuing their regular tasks.

According to the local professionals, the network mostly worked on getting the members across different sectors to know each other. Many attendants at these working group meetings said that they had met professionals from other institutions in the network that they had not known before. Furthermore, in some cases, they intended to contact them at a later time. However, no interviewee named a specific case where the network had helped them when working directly with their clients. Also, only in one case an interviewed professional stated that a working group meeting had resulted in a bilateral cooperation. Additionally, some attendants commented that the process of getting to know each other was taking too long and that they would prefer to start working on specific tasks.

“If you are meeting just for the sake of meeting, that is not enough in the long run, at least for me.” *(44401)*.

Due to the COVID-19 pandemic, working group meetings of the local network took place virtually. While some professionals stated that this format made it easier to attend the meetings, others said that output-oriented work would be impeded under these conditions.

“Discussing which topics to work on together (…) is currently more difficult with corona [the COVID-19 pandemic]. Everything is just virtual at the moment.” *(89616)*.

Many interviewees agreed that the network was not yet operating as a production network that created palpable output. Beside the COVID-19 pandemic, this was mainly explained with the early development status of the Freiham district. Many institutions relevant for the network were not active in the area yet, and only few residents had already moved in. According to some interviewees, this made it hard to find specific tasks to work on. Furthermore, despite Freiham being a new district, the existing Munich networking structure called REGSAM (“Regionales Netzwerk für Soziale Arbeit”, Regional Network for Social Work*)* with its focus on the social sector was already active in Freiham. It was expressed to be a challenge to communicate the advantage of a new network to professionals. Furthermore, stakeholders of Präventionskette Freiham and the REGSAM network had to coordinate with each other to avoid conflicting responsibilities.

In the focus group, the cooperation between the three municipal departments in the advisory group was generally described as pragmatic and trusting. However, members of the advisory group reported problems with creating interest for the Präventionskette within the municipal administration. Furthermore, as part of the administration, members of the advisory group were not allowed to communicate directly with political decision makers about the Präventionskette. Only the heads of the municipal departments would be allowed to engage directly with political decision makers. However, their engagement for the network was unclear for the members of the advisory group.

“The leadership here (…) knows about the Präventionskette, I guess, but to what extent she is considering it when making decisions, is completely unclear to me.” *(70534)*.

#### Facilitators and barriers to implementation

We identified seven core topics that could work as potential facilitators or barriers for a successful implementation of the intervention. An overview can be found in Table 1.

##### Resources

Availability of resources was often named to be an important factor that could be a facilitator or barrier. Interviewees expressed that both the network coordinators and local institutions needed sufficient funding and, related to that, sufficient human resources to do the work required for running the network. In particular, local professionals stated that limited temporal capacities were a barrier for a stronger engagement with the network. This was perceived to have been amplified by the COVID-19 pandemic, which placed additional demands on the workforce from the health care and educational sectors, meaning that time could not be invested in networking.

##### Administrative and political support

A long-term engagement by the municipal administration and by municipal politics was considered a major contributor to the success of the intervention by many interviewees. During the study period, the network coordinators´ funding was only secured for a limited period of time, and the long-term perspective remained uncertain. Interviewees commented that a lot of resources had to be invested by the network coordinators and the advisory group to secure continued support and funding. These resources could otherwise have been spent on building the network. Local professionals in Freiham expressed the concern that, without the network coordinators, the networking processes that had been happening so far might come to an end. Furthermore, some raised the concern that the uncertain future of the network´s funding might prevent some other local actors from engaging.

##### Network coordinators

The persons responsible for coordinating the network were considered another important facilitator for the success of the intervention. According to the interviewees, the persons have to be well-connected, be able to integrate the different working cultures of the members of the network and be aware of the specific context in Freiham to set meaningful priorities for the activities of the network. It was also considered an advantage that the network coordinators were not employees of the city administration, but came from another institution, as it allowed them to act more independently.

##### Network-internal processes

An atmosphere of trust between the members of the local network, transparent communication processes as well as a participatory working culture were identified as facilitators for a successful implementation. Interviewees outlined the need to involve not only members of the local network in decision making processes, but also the target groups of the intervention and other actors in the district of Freiham that are external to the network, such as voluntary organizations and elderly citizens or privately organized day care centers. Furthermore, professionals expressed uncertainty about their roles and tasks within the network, affecting their engagement negatively.

##### Trans-institutional cooperation

Integrating actors from different sectors into the local network was considered a facilitator for the functioning of the network, as it allowed the network to cover all areas of life for children and adolescents. Additionally, it was stated that it enabled single members of the network to change perspectives and to gain more insights into the needs of the target groups. For trans-institutional cooperation, it was considered important that the members of the network should develop a shared vision with regards to the goals of the intervention. However, barriers to this trans-institutional cooperation were also expressed, mainly that professionals from different institutions often had differing needs that might be hard to integrate. For instance, they were used to different work routines, e.g. when leading discussions or making decisions, according to our interviewees. Furthermore, due to data protection regulations, some professionals said that they were hesitant to talk about specific cases within the working group meetings of the network, as regulations from their institution forbade them to do so.

**Table 1:** Facilitators and barriers to the implementation of Präventionskette Freiham identified in the interviews with the network coordinators and members of the local network and in the focus group with members of the advisory group

Topic	Facilitator	Barrier	Signifying quote
Resources	• Sufficient funding for network coordinators • Sufficient resources (workforce, funding) for local institutions	• Insufficient funding for network coordinators • Insufficient resources (workforce, funding) for local institutions	“Time is always a barrier, for everyone. We experience this at schools currently, too. So, a second meeting is scheduled. But then, something more important comes up or someone gets ill and then the stakeholders do not come. Especially in day-to-day business, everything else is always more important or urgent.” *(86744)*
Administrative and political support	• Long-term engagement by municipal administration and politics	• Spending workforce to secure support • Uncertain perspective of network coordinators preventing local stakeholders from engaging in network	“For many people, how much energy and time they put into networking depends on funding, if there is no funding after a year, they will probably hold back a bit.” *(26626)*
Network coordinators	• Well-connected at municipal and district level • Ability to integrate different working cultures in the network • Agenda-setting • Independence from municipal administration		“That is certainly one task. Shaping the willingness to participate, finding the right topics that institutions cannot do just as well or better on their own (…)” *(47134)*
Network-internal processes	• Trust • Continuity • Transparent communication • Participatory approach (professionals and target groups)	• Uncertainty about roles • Uncertainty about tasks	“Or building personal, trusting contact with the institutions. (…) Then you know that you can contribute to the group, that you are taken seriously in the group.” *(47134)*
Trans-institutional cooperation	• Integrating actors from different sectors into the network • Change of perspectives • Vision of shared goals	• Differing needs of institutions • Data protection regulations	“Independent of data protection, (…) the thought ´this is my client` is very strong in individual counselling of families. (…) You don’t talk about them outside, not even anonymously, and that’s how I know it.” *(26626)*
Perceived benefits to network members	• Increasing individual networks • Information	• Uncertainty about benefits	“So, if I go there I would need some profit from it. Why do I go there? It would need to provide me some form of help. Or I would need to get some information in advance.” *(36651)*
Output	• Sustainability	• Early development status of residential area • Pursuing short-term outcomes	“If people were already living in the area, then you could identify existing issues and work on them. I don’t know how to say this correctly, but if there are no real problems, challenges in the area yet, then you might be tempted to search for or create some.” *(86744)*

##### Perceived benefits to network members

Many members of the local network stated that the network would have to offer benefits to its members to make an engagement attractive. Interviewees considered being able to expand their personal networks and getting access to information that would otherwise be hard to obtain as the most important benefits. Local professionals wanted to specifically receive information from within the city administration as there were many uncertainties about the future development of the Freiham district.

##### Output

Participants emphasized the need to create sustainable outputs to achieve structural benefits for the residents instead of using resources towards short-term goals. A barrier to developing long-term output was the perceived need to show quick successes to ensure sustained support from policy-makers and the municipal administration. The early development status of the Freiham district was identified as a general barrier for creating meaningful output. With only few citizens living in the area, interviewees described a lack of real tasks to work on and felt unsure about which activities would become most relevant in the future. One local professional was concerned that these circumstances might lead the network to engage in short-term unsustainable projects, just to be able to showcase output.

### Results for the ego-centered network maps

**Fig. 4:**
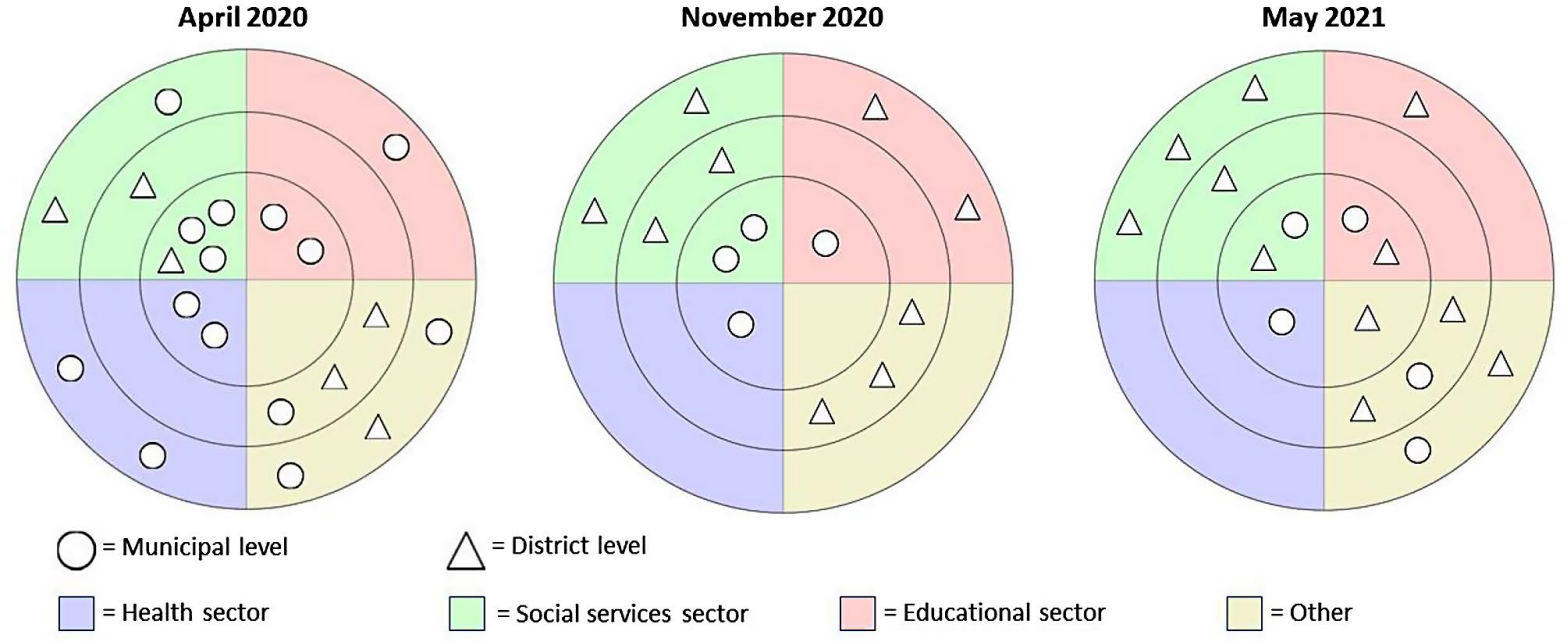
Ego-centered network maps showing the most important collaborators for the network coordinators at the respective time (inner circle: “strong cooperation”; middle circle: “medium cooperation”; outer circle: “weak cooperation”)

A total of three network maps were drawn by the network coordinators: in April 2020, November 2020 and May 2021 (Fig. 4). As there was a change in the position of the network coordinator in August 2020, the two subsequent network maps were drawn by a different person. This break is represented in the network maps, where the number of included actors dropped from 20 in April 2020 to 13 in November 2020. Over the period of data collection, the number of actors working in Freiham named in the network maps increased from six (30%) in April 2020 to nine (69%) in November 2020 and eleven (69%) in May 2021. When considering only the actors with “strong cooperation”, actors at the municipal level made up the majority during most of the time (7 out of 8 in April 2020; 4 out of 4 in November 2020). This slightly changed at the time of the last network map (3 out of 6 in May 2021). None of the network maps identified an actor from the health sector working locally in the district of Freiham. Overall, most actors that could be assigned to one of the three sectors were from the social services, with only few being part of the health and educational sectors. A lot of actors could not be located to one of the three sectors, mainly among those working in the district. While they were important and well-connected actors in the Freiham area and therefore relevant for the network coordinators, their responsibilities could not be reduced to the health, educational or social sector alone.

### Results for the survey with members of the local network

Of the 67 members of the local network invited to participate per e-mail, 25 opened the link to the digital survey. Of these, six dropped out of the survey before or directly after answering the first question and were excluded from the analysis. Among the 19 participants we included, 17 filled out the survey completely and two incompletely.

Overall, 15 participants had joined the regular working group meetings of the network at least every second time since September 2021, three had participated less regularly. A majority of the 19 participants agreed with the statement that they were satisfied with the working group meetings of the local network and that they were able to expand their personal networks through these meetings (Table 2). Opinions were less positive regarding whether participating in the network meetings had been helpful for their daily work or whether it had increased their expertise.

Four participants stated that some of their expectations regarding the network coordinators had not been fulfilled. In the open answer option, participants expressed that they wanted more support for working on specific projects. Furthermore, four participants suggested changes to the local network’s work mode, e.g. reducing the number and length of network meetings and building stronger connections between the local network meetings and the municipal administration. On the other hand, participants noted that the involvement of individual institutions with the network and the exchange of district-related information should be kept as integral parts of the meetings. When asked for relevant missing actors, participants named schools and day-care centers, but also private resident organizations and churches.

**Table 2:** Results of the survey with members of the local network of Präventionskette Freiham regarding the regular working group meetings. The survey was conducted in August and September 2022

Item			I disagree (n)		I rather disagree (n)		I neither agree, nor disagree (n)		I rather agree (n)		I agree (n)	Total (n)
Overall, I am satisfied with the working group meetings			0		1		6		5		7	19
Participating in the meetings is helpful for my daily work			1		5		6		4		3	19
The meetings increased my expertise			0		4		6		8		1	19
I was able to expand my network through the meetings			1		3		3		4		8	19

## Discussion

In this study, we investigated the implementation process of the intervention Präventionskette Freiham in a new residential development area. We found that the implementation only made slow progress in its early stages and that the members of the local network found it difficult to work on refining the support infrastructure for children and families. In the interviews and the focus group, several explanations for this were presented. While the COVID-19 pandemic impeded networking processes, many participants felt that working in the context of a new residential development area likely constituted a more important factor. As potential facilitators or barriers for the success of the intervention, we identified availability of resources, support from the municipal administration and political decision-makers, the network coordinators as key actors, network-internal processes, trans-institutional cooperation, perceived benefits to members and the ability of the network to create output they considered sustainable. Ego-centered network maps by the network coordinators show that the network included few professionals from the health and educational sectors, a finding that was confirmed in the interviews and the survey. In both the interviews and the survey, members of the local network stated that they had been able to widen their network by participating in the Präventionskette, while the relevance of the network for their daily work had been minor.

Our results are mostly congruent with previous research on implementing integrated community-based interventions. Availability of sufficient resources is stated as a key facilitator in the Bergen Model of Collaborative Functioning by Corbin et al. [38] and in a synthesis model for intersectoral collaboration in municipal health promotion and prevention by Quilling et al. that was designed based on a scoping review on existing models [39]. Resources were also one of the most common success factors for intersectoral partnerships for health equity, as found in a scoping review by Chircop et al. [40]. Similarly, support from political decision-makers has been identified as an important success factor in the review by Quilling et al. [39], in an evaluation of the Zwolle Healthy City approach, an integrated community-based intervention for tackling socioeconomic health inequalities in the Netherlands [41], and in previous research on Präventionsketten in different German municipalities [29, 42]. In the case of Präventionskette Freiham, interviewees felt unsure about how much the intervention was supported by political decision-makers and the top-level administration. This constituted a source of uncertainty among partners, as this support was directly associated with the question whether the funding of the network would be continued. Transparent communication [39, 41, 43] and the need for leadership [17, 39, 40, 43] have also been widely acknowledged as positive factors in previous research to facilitate participation of institutions and professionals in intersectoral public health interventions.

Integrating partners from different sectors and with differing interests in a network using a shared goal has often been identified as a facilitator for intersectoral approaches [17, 38–40]. This was also expressed in our interviews. However, several professionals said that they felt unsure about their tasks and roles within the network, leading them to take on a more observing rather than a proactive role. At the same time, interviewees said that the network found it difficult to integrate professionals from the educational and health sectors, which was mirrored in the network maps drawn by the network coordinators. Previous studies have found that specifically engaging schools in intersectoral partnerships can be a challenge [44–46], independent of the challenges of a pandemic. In a Danish survey exploring the experiences of educational consultants with municipal health promotion programs in schools, participants stated that teachers tended to consider these activities an additional burden [45], an opinion that was also expressed in our interviews as a barrier to a greater commitment by the educational sector.

While most of our results align with previous research on public health interventions that involve intersectoral networks in general, some results seem to be specific to the context of a new residential development area. A limited period of funding for the network was considered a barrier to creating output, amplified by the fact that both institutions and residents were still not fully present in the area. When planning new interventions or adapting already existing interventions to these settings, the additional time to build a network and to create output needs to be considered by funders and decision-makers. Furthermore, despite Freiham being a new residential development area, the system in which Präventionskette Freiham was implemented was not blank, as there were already other networking structures operating in the area. Therefore, even in newly developing districts, stakeholders planning or implementing an intervention should analyze the existing structures at different levels to assess potential synergies and conflicts. In the Bergen Model of Collaborative Functioning, context factors, such as negative communication, unclear roles and negative leadership, can be a source of antagony – negative results that may decrease an intersectoral partnership’s abilities to reach its goals [43].

In multiple instances the intervention and the specific context of a new residential development area interacted with each other. As many institutions were not yet present in the area and only few residents had already arrived in Freiham, the network found it hard to evaluate which to prioritise, and a lot of time had to be spent on professionals getting to know each other. On the other hand, the intervention was able to implement a networking structure in the area that otherwise might not have developed. However, within the restricted time of the process evaluation, the effect on the support infrastructure beyond that was limited. These findings highlight the importance of applying a complex systems perspective when planning an intervention, i.e. taking the existing system into consideration and exploring how the system and the intervention might interact [47], may help to avoid antagonistic effects and increase chances for a successful implementation.

### Strengths and limitations

This study has several strengths. By pursuing a mixed methods approach, we were able to triangulate results of the qualitative interviews, the focus group, network maps and the survey to increase the scope and the validity of our research. As discussed before, the results from these different approaches align with each other. Furthermore, rooted in a complexity perspective and informed by a logic model, we explored the perspectives of multiple stakeholders from different sectors at multiple time points. This allowed us to obtain broad as well as deep insights into the implementation process. Data collection and analysis of the qualitative interviews and the focus group were conducted by a team of experienced researchers and described following the COREQ guidelines (“Consolidated criteria for reporting qualitative research”) [48]. Qualitative content analysis was conducted in an iterative process by a team of three researchers. All conflicts during this process were resolved within the team before progressing to the next step, increasing the validity of the results we obtained.

There are also limitations to this study. While our mixed methods approach allowed us to triangulate our results, each of the individual data sources has its limitations. The network maps were not created by the same person, but by two different individuals. Therefore, changes in the network over time visualized through these maps may not be due to the actual developments, but due to the change in the person who drew them. Furthermore, we conducted this study at an early stage of the implementation of Präventionskette Freiham. While this allowed us to gain very specific insights into the initial conditions of the implementation process, we cannot extrapolate from our findings to make statements about what facilitators and barriers an intersectoral network in a new residential development area may face at a later stage with more established structures and working conditions. Regarding data collection, while using the network coordinators as gatekeepers facilitated access to members of the local network, it may be possible that professionals with a positive attitude towards the network coordinators were more willing to agree to be interviewed, leading to a selection bias. This sampling strategy may have led to a trend towards socially desirable answer behavior. The same holds true for the survey with members of the local network, where the network coordinators sent out invitations and reminders. In the focus group with employees from the municipal administration, all participants were members of the advisory group of Präventionskette Freiham. While we were able to gain insights from key stakeholders of the intervention, we did not investigate the perspectives of employees that were less engaged with the intervention and may have differing views on facilitators and barriers.

One member of the research team (MC) was part of the advisory group of Präventionskette Freiham. Other members of the research team (CJS, ER, JB), including the first author (SV) who conducted the interviews, participated in several meetings with stakeholders of the intervention, to present research findings and discuss implications for the further implementation of the intervention. Thus, the researchers did not take the role of external observers of the implementation process, but themselves became actors in the intervention. It is likely that they were perceived in this role by others, too, mainly by the professionals that were interviewed, and that this perception has influenced the answers given during data collection.

## Conclusions

In this study, we identified sufficient resources, funding, support by the city administration and local politics and perceived benefits for members as key facilitators for implementing an integrated community-based intervention in a new residential development area. However, we found the absence of institutions and residents during the early development stages of the district to be a barrier for the network to operate as intended. This highlights the need for long-term commitment and investment by all concerned stakeholders as well as realistic time frames and expectations. Decisionmakers in municipal politics and administrations should consider these when planning and conducting intersectoral public health interventions in new residential development areas. As a process evaluation, this research aimed at investigating the implementation process of the intervention Präventionskette Freiham. Whether implementing intersectoral networks while building a new district represents an effective approach to provide health equity for children and adolescents, will be the subject of future research.

### Electronic supplementary material

Below is the link to the electronic supplementary material.

**Supplementary Material:** Interview guides and questionnaire of the survey with network members

## Data Availability

The datasets used and/or analysed during the current study are available from the corresponding author on reasonable request.
