# Supplementary material for: Process evaluation of an integrated community-based intervention for promoting health equity in children in a new residential development area
Source: Arch Public Health. 2024 Feb 6;82:19. doi: 10.1186/s13690-024-01246-z (PMC10845767; doi:10.1186/s13690-024-01246-z)
Supplement: Supplementary file 1 — Supplementary Material: Interview guides and questionnaire of the survey with network members [file 13690_2024_1246_MOESM1_ESM.docx]

**Supplementary Material**

**Process evaluation of an integrated community-based intervention for promoting health equity in children in a new residential development area**

Authors: Stephan Voss^1,2^, Julia Bauer^,2^, Caroline Jung-Sievers^1,2^, Graham Moore^3,4^, Eva Rehfuess^1,2^, Valerie Zu Rhein^1,2^, Michaela Coenen^1,2^

^1^ Chair of Public Health and Health Services Research, Institute for Medical Information Processing, Biometry, and Epidemiology (IBE), Faculty of Medicine, LMU Munich, Elisabeth-Winterhalter-Weg 6, 81377 Munich, Germany

*^2^* Pettenkofer School of Public Health, Munich, Germany

^3^ Centre for Development, Evaluation, Complexity and Implementation in Public Health Improvement (DECIPHer), School of Social Sciences, Cardiff University, Cardiff, UK

^4^ Wolfson Centre for Young People’s Mental Health, Cardiff University, Cardiff, UK

# Appendix 1: Interview guides for interviews with network coordination, local professionals and focus group with advisory group

## Interview Guide Network Coordination

| **Block 1: Current role** | | |
| --- | --- | --- |
| **Key question** | **Specific question** | **Checklist** |
| Can you briefly describe our role within Präventionskette Freiham? | Which institution do you belong to?  Can you describe what your tasks are at Präventionskette Freiham?  Can you say how much time you spend working for Präventionskette Freiham on average per week?  How much time do you spend working in Freiham? | - Tasks - Institution - Capacities |
| **Block 2: Current activities** | | |
| **Key question** | **Specific question** | **Checklist** |
| Can you describe the projects you are currently working on as part of Präventionskette Freiham? | Follow-up questions on individual activities/projects:   - Can you describe the activities in more detail? - What are the objectives of the project? - What are the target groups of the project? - Can you describe how these target groups take up these offers? - What are stakeholders involved in this project? - What is your role within this project? - From whom did the initiative for this project come? - How has the COVID-19 pandemic affected the project?   Are there any other projects being worked on within Präventionskette Freiham, independent of the network coordination? | - Record activities with their respective: - Actors - Activities - Goals - Acceptance of target groups |
| **Block 3: Target groups** | | |
| **Key question** | **Specific question** | **Checklist** |
| Can you describe what kind of contacts you are currently having with residents? | How well do you reach residents with current services?  Are there projects/activities in planning that involve residents?  In your opinion, where are the greatest gaps in demand?  In reaching which target groups do you see a need to catch up?  What can be done to reach them better? | - Assessment of reaching target groups - Gaps in demand - Planned and current measures to reach target groups - Contact with target groups |

- **Pause in interview and start of drawing the network map (every second interview):**

| **Block 4: Network with actors** | | |
| --- | --- | --- |
| **Key question** | **Specific question** | **Checklist** |
| How would you describe the current state of the network? | How do you feel about the cooperation with the advisory group?  How would you describe the networking process with local professionals?  With which persons and institutions do you currently cooperate within Präventionskette Freiham? (If institution named: Which persons there are relevant for the cooperation?)  In your opinion, at which points does the cooperation work particularly well?  At which points does it work less well in your opinion?  Why is this the case?  From your point of view: Which actors, who are not currently involved, should be involved?  Which partners are currently most important for the success of Präventionskette Freiham and why?  Are there cooperations that have been formed as part of Präventionskette Freiham but in which you are not involved? | - Degree of networking - Currently relevant actors - Currently not involved actors - Relevance with reasonings |
| **Block 5: Review and outlook** | | |
| **Key question** | **Specific question** | **Checklist** |
| How would you describe the development since the last interview? | From your point of view, what positive developments have occurred during the establishment of Präventionskette Freiham?  From your point of view, what obstacles did you encounter in implementing Präventionskette Freiham?  Specifically, how did the COVID-19 pandemic impact your work?  Have there been other areas of progress? | - Assessment of implementation process - Facilitators - Barriers |
| Can you describe what you consider to be the most important tasks for Präventionskette Freiham in the next 6 months? | What activities are currently being planned?  In which areas is there the greatest need for new services?  Specifically, what impact do you expect the COVID-19 pandemic to have on future implementation?  In your opinion, which actors still need to be involved?  Why are they not participating so far?  What do you see as the biggest problems at the moment? | - Future needs - Planned projects - Facilitators - Barriers |

## Guide focus group advisory group

| **Block 1: Role of advisory group** | | |
| --- | --- | --- |
| **Key question** | **Specific question** | **Checklist** |
| Can you briefly introduce yourself by name and position within the advisory group? And since when you have been part of the advisory group? |  | - Description of participants - Perceiving of own role |
| **Block 2: Cooperation within the advisory group** | | |
| **Key question** | **Specific question** | **Checklist** |
| How have you perceived the cooperation within the advisory group so far? | Were there any key moments during your work in the advisory group that particularly stuck in your memory?  How did the COVID-19 pandemic affect the work in the advisory group?  * How do you think this cooperation has evolved since the start of the advisory group?  * How have your expectations of the work in the advisory group developed during this time?  What do you think have been problems for the cooperation within the advisory group?  In your opinion, what factors have been facilitators for the cooperation within the advisory group?  Can you give examples for that?  What are your ideas for the future work of the advisory group?  Is there anything else you would like to say about this? | - Current state of cooperation - Summary of cooperation so far - Facilitators/barriers - Expectations regarding future cooperation |
| **Block 3: Cooperation with network coordination** | | |
| **Key question** | **Specific question** | **Checklist** |
| How would you describe the current cooperation with the network coordination? | How did the cooperation between the advisory group and the network coordination develop from your point of view?  Were there any particular milestones in your opinion?  How do you envision the cooperation with the network coordination in the future?  In your opinion, what are facilitators for the cooperation between the advisory group and the network coordination?  In your opinion, what are barriers for the cooperation between the advisory group and the network coordination? | - Cooperation with network coordination - Development of cooperation - Expectations regarding future cooperation - Facilitators/barriers |
| **Block 4: Embeddedness in municipal administration** | | |
| **Key question** | **Specific question** | **Checklist** |
| Can you describe how Präventionskette Freiham is anchored in the municipal administration outside the monitoring group? | Can you say to what extent the municipal departments and the administration also cooperate regarding Präventionskette Freiham outside the advisory group?  Can you give examples?  What are facilitators for cooperation between the departments?  What are barriers to cooperation?  Where do you see a need for improvement?  How Präventionskette Freiham perceived in the municipal administration?  Are there actors in the administration that are particularly engaged in Präventionskette Freiham?  Are there actors that you think should be more engaged in Präventionskette Freiham?  How has the COVID-19 pandemic affected cooperation between municipal departments regarding Präventionskette Freiham?  Is there anything else you would like to say about this? | - Position of Präventionskette Freiham in the municipal administration - Cooperation within municipal administration - Facilitators/Barriers |
| **Block 5: Cooperation with local professionals** | | |
| **Key question** | **Specific question** | **Checklist** |
| Can you describe how you assess the development of the network in the district? | How do you feel about the state of networking with local professionals?  In your opinion, what are the most important institutions or actors for the Präventionskette in Freiham?  What are important factors for the network to be successful in the district?  What are potential challenges?  Can you say which of your expectations for the network in the district have been fulfilled?  Can you say which of your expectations have not been fulfilled?  How do you feel about the state of cooperation with local professionals?  Can you give examples where cooperation has worked particularly well in the past?  Can you give examples where cooperation has worked less well in the past?  What were the reasons?  What lessons do you think can be learned from these examples?  How does the COVID-19 pandemic affect cooperation in the district?  Is there anything else you would like to say about this? | - Network with local professionals - Facilitators and barriers - Expectations regarding networking process |
| **Block 6: Future development** | | |
| **Key question** | **Specific question** | **Checklist** |
| What do you consider to be the most important tasks for Präventionskette Freiham in the next months? | Where do you see Präventionskette Freiham in one year?  What do you see as specific next steps that need to be taken in Präventionskette Freiham?  How do you see the future role of the advisory group?  Is there anything else you would like to say about this? | - Future developments - Future projects - Future of advisory group |

## Interview guide local professionals (1st interview)

| **Block 1: Role in Freiham** | | |
| --- | --- | --- |
| **Key question** | **Specific question** | **Checklist** |
| Can you briefly describe your professional activity? | From which areas do the children and families come from you are working with? | - Field of activity - Contact with target group |
| **Specific 2: Role of Präventionskette Freiham** | | |
| Can you describe what Präventionskette Freiham is in your own words? | Can you describe the goals of Präventionskette Freiham?  Which connections do you see between your own work and Präventionskette Freiham?  Which priorities should Präventionskette Freiham set in your opinion? | - Knowledge of Präventionskette   - Tasks/Goals   - Structure - Relevance for own work |
| **Block 3: Experience with network coordination** | | |
| **Key question** | **Specific question** | **Checklist** |
| Can you tell us about the experiences you have made with the network coordination of Präventionskette Freiham so far? | Can you tell how the contact with the network coordination of Präventionskette Freiham came about?  In which concrete activities (actions, participation in meetings/video conferences) have you been involved in the Präventionskette so far?  Can you describe the project/meeting in more detail?  Can you say what the goal or goals of this meeting/project were?  Who took the initiative to do so?  Who was involved in it?  What was your motivation for being involved here?  Can you say what you rather liked about it?  Can you say what you did not like so much?  Were you also involved in activities in the district that were not initiated by the network coordination of the Präventionskette?  Are there any other points you would like to say about your experience with the network of the Präventionskette so far? | Activities to date   - Contact - Course - Goals - Personal motivation - Personal assessment |
| **Block 4: Needs for cooperation** | | |
| **Key question** | **Specific question** | **Checklist** |
| To what extent do you see a need for cooperation with other institutions or actors in your work? | How is the cooperation with other institutions or actors in Freiham going so far?  How do you perceive the cooperation among the institutions in Freiham?  What projects or measures would be useful in this regard from your point of view? | - Need for cooperation - Suggestions for projects |
| **Block 5: Needs of residents** | | |
| **Key question** | **Specific question** | **Checklist** |
| Which needs do you see for children and their families in Freiham? | When you think about your work in your institution: Which problems of children, adolescents, pregnant women and families in Freiham are you aware of?  Have you also noticed corresponding issues (or problems/needs) outside of your work?  What can be done about these problems from your point of view? | - Needs of target groups - Options for action |
| **Block 6: Ideas and expectations for future cooperation** | | |
| **Key question** | **Specific question** | **Checklist** |
| What are your personal expectations regarding the cooperation with Präventionskette Freiham? | Where do you see the benefit for yourself or your institution in participating in the network of Präventionskette Freiham?  On which topics do you see a need for cross-institutional cooperation?  In what way would this be beneficial?  What does collaboration currently look like here?  Do you plan to participate in network of Präventionskette Freiham in the future?  In what kind?  Also, looking back at your experience so far: Is there anything you would like to be different in the future?  In your view, are there any issues that urgently need to be addressed? | - Plans for future cooperation - Facilitators and barriers - Needs for cooperation - Ideas for improvement |

## Interview guide local professionals (2nd interview)

| **Block 1: Situation since last interview** | | |
| --- | --- | --- |
| **Key question** | **Specific question** | **Checklist** |
| Can you tell me what the challenges have been for your work in the past months? | What has been the impact of the COVID-19 pandemic?  What has the cooperation with other actors looked like? | - Influence of COVID-19 pandemic - Current state of cooperation |
| **Block 2: Experiences with Präventionskette Freiham** | | |
| **Key question** | **Specific question** | **Checklist** |
| Can you tell us which experiences you have made with the network coordination of Präventionskette Freiham in the last months? | Can you tell how the contact with the network coordination of Präventionskette Freiham came about?  In which concrete activities (actions, participation in meetings/video conferences) have you been involved in the Präventionskette so far?  Can you describe the project/meeting in more detail?  Can you say what the goal or goals of this meeting/project were?  Who took the initiative to do so?  Who was involved in it?  What was your motivation for being involved here?  Can you say what you rather liked about it?  Can you say what you did not like so much?  How did you experience these activities compared to the first ones within Präventionskette Freiham?  Are there any other points you would like to say about your experience with the network of the Präventionskette so far? | Activities to date   - Contact - Course - Goals - Personal motivation - Personal assessment |
| **Block 3: Motivation for cooperation** | | |
| **Key question** | **Specific question** | **Checklist** |
| To what extent do you see a need for cooperation with other institutions or actors in your work? | How is the cooperation with other institutions or actors in Freiham going so far?  How do you perceive the cooperation among the institutions in Freiham?  What projects or measures would be useful in this regard from your point of view? | - Need for cooperation   Suggestions for projects |
| **Block 4: Needs of target groups** | | |
| **Key question** | **Key question** | **Key question** |
| Which needs do you see for children and their families in Freiham? | What needs do you see for children and young people and their families in Freiham, beyond your work?  Have you also noticed corresponding issues (or problems/needs) outside of your work?  In light of the COVID-19 pandemic, what are important actions for the near future?  What issues will become important in Freiham in the future?  What can be done about these issues in your view?  How could the prevention chain be of use here? | - Needs of target groups - Options for activities - Role of Präventionskette |
| **Block 5: Ideas and expectations for future cooperation** | | |
| **Key question** | **Specific question** | **Checklist** |
| What are your plans regarding cooperating with the network coordination of Präventionskette Freiham in the future? | In what form?  Looking at your previous experiences: Is there anything you would like to see happen differently in the future?  In your view, are there any issues that urgently need to be addressed? | - Plans for future cooperation - Facilitators and barriers - Needs for cooperation - Ideas for improvement |
| **Block 6: Challenges and risks** | | |
| **Key question** | **Specific question** | **Checklist** |
| In your opinion, what is important for the project to be a success? | What are facilitators?  What are barriers?  What are opportunities for networking in the district? | - Facilitators - Barriers |
| **Block 7: Summary** | | |
| **Key question** | **Specific question** | **Checklist** |
| Summing up, how would you describe your experience with Präventionskette Freiham? | How has cooperating with Präventionskette Freiham benefitted you for your personal work?  What can be done to improve the willingness to cooperate?  What are points that you feel are rather positive?  What are points that you feel are less positive?  How do you feel about the concept of commitment?  Is there anything else you would like to say on the subject of Präventionskette Freiham? | - Summary personal perspective on Präventionskette Freiham |

# Appendix 2

Präventionskette Freiham: Survey with members of the network

Dear network members,

Thank you for participating in this survey. It will take about 10 to 15 minutes of your time.

In cooperation with the network management of Präventionskette Freiham, this survey is conducted by the Chair of Public Health and Health Services Research at LMU Munich. The purpose of the survey is to obtain your feedback on the development of the Präventionskette and your ideas for further development.

Your participation is voluntary. The survey is also intended to be anonymous. Therefore, please make sure that you do not provide any information about yourself or other persons or institutions in the free input fields and that your answers cannot be traced back to you personally.

**Meetings of the working groups**

**In which working group of Präventionskette Freiham are you a member?**

*Please select one of the following answers:*

- Working group 0-6
- Working group 6-17+
- Both working groups (0-6 and WG 6-17+)
- None of them

**How often have you attended the regularly scheduled meetings of the working group 0-6 since September 2021?**

*Please select one of the following answers:*

- Less frequently than every second time
- More often than every second

**How often have you attended the regularly scheduled meetings of the working group6-17+ since September 2021?**

*Please select one of the following answers:*

- Less frequently than every second time
- More often than every second

**How often have you attended the regularly scheduled meetings of the two working groups since September 2021?**

*Please select one of the following answers:*

- Less frequently than every second time
- More often than every second

**How do you feel about the frequency of the working group meetings?**

*Please select one of the following answers:*

- Too seldom
- Rather too seldom
- Just right
- Rather too often
- Too often

**How do you feel about the duration of the working group meetings?**

*Please select one of the following answers:*

- Too short
- Rather too short
- Just right
- Rather too long
- Too long

**How much do you agree with the following statements?**

Please select the applicable answer for each item**:**

|  | I disagree | I rather disagree | Neither, nor | I rather agree | I agree |
| --- | --- | --- | --- | --- | --- |
| Overall, I am satisfied with the working group meetings. |  |  |  |  |  |
| Participating in the meetings is helpful for my daily work. |  |  |  |  |  |
| The meetings have expanded my expertise. |  |  |  |  |  |
| I was able to expand my network through the meetings. |  |  |  |  |  |

**If you would like, please feel free to describe in more detail here how the meetings were helpful to you and/or why you were satisfied or dissatisfied with them:**

Please enter your response here:

When responding, please be careful not to provide any information that could be used to identify you or other persons or institutions.

**What changes would you like to see?**

Please enter your answer here:

When responding, please be careful not to provide any information that could be used to identify you or other persons or institutions.

**Which aspects of the meetings or the previous works of the network should in your opinion be retained?**

Please enter your answer here:

**What were these expectations?**

Please enter your answer here:

When responding, please be careful not to provide any information that could be used to identify you or other persons or institutions.

**Development of the network**

Are there any expectations you have of the network management or the working group meetings that have not yet been met?

*Please select only one of the following answers:*

- Yes
- No

**Would you like to see changes for the working group meetings and the network?**

*Please select only one of the following answers:*

- Yes
- No

**In your opinion, are there still actors from important areas missing from the working group meetings or in the network?**

*Please select only one of the following answers:*

- Yes
- No

**Do you have any further feedback?**

Please enter your answer here:

When responding, please be careful not to provide any information that could be used to identify you or other persons or institutions.

**What can we as Präventionskette Freiham do to improve the situation of children, young people and families in the neighborhood?**

Please enter your answer here:

When responding, please be careful not to provide any information that could be used to identify you or other persons or institutions.

**Which areas are these?**

Please enter your answer here:

When responding, please be careful not to provide any information that could be used to identify you or other persons or institutions.

Thank you for your participation! You can now close the survey.
